# Transformation of *Helicobacter pylori* into Coccoid Forms as a Challenge for Research Determining Activity of Antimicrobial Substances

**DOI:** 10.3390/pathogens9030184

**Published:** 2020-03-04

**Authors:** Paweł Krzyżek, Rossella Grande

**Affiliations:** 1 Department of Microbiology, Faculty of Medicine, Wroclaw Medical University, 50-368 Wroclaw, Poland; 2 Center for Aging Science and Translational Medicine (CeSI-MeT), Via Luigi Polacchi, 11, 66100 Chieti, Italy; r.grande@unich.it; 3 Department of Pharmacy, University “G. d’Annunzio” of Chieti-Pescara, Via dei Vestini, 31, 66100 Chieti, Italy

**Keywords:** *H. pylori*, coccoid forms, morphological transformation, antibacterial therapies

## Abstract

Morphological variability is one of the phenotypic features related to adaptation of microorganisms to stressful environmental conditions and increased tolerance to antimicrobial substances. *Helicobacter pylori*, a gastric mucosal pathogen, is characterized by a high heterogeneity and an ability to transform from a spiral to a coccoid form. The presence of the coccoid form is associated with the capacity to avoid immune system detection and to promote therapeutic failures. For this reason, it seems that the investigation for new, alternative methods combating *H. pylori* should include research of coccoid forms of this pathogen. The current review aimed at collecting information about the activity of antibacterial substances against *H. pylori* in the context of the morphological variability of this bacterium. The collected data was discussed in terms of the type of substances used, applied research techniques, and interpretation of results. The review was extended by a polemic on the limitations in determining the viability of coccoid *H. pylori* forms. Finally, recommendations which can help in future research aiming to find new compounds with a potential to eradicate *H. pylori* have been formulated.

## 1. Introduction

*Helicobacter pylori* is a bacterium that colonizes over 60% of people in the world [1]. The presence of this microorganism, however, is not neutral for the host, and is accompanied by the development of gastritis, which over the years may progress to gastric ulcers or cancers [2]. The appearance of these diseases is driven by an expression of a wide range of virulence factors, both adhesins and toxins [3]. Hence, the latest Maastricht V recommendations pointed to the necessity for the eradication of *H. pylori* infections, regardless of the presence of disease symptoms [4].

The prevalence of *H. pylori* and its impact on human health have contributed to the high intensity of research focusing on epidemiology, diagnostics, and treatment of this microorganism [5,6,7]. Because nowadays antibiotics are the only accepted form of combating *H. pylori*, the resistance of this microorganism to these medications is an alarming problem [8]. It has been observed that the frequency of resistance to three out of the five commonly used antibiotics (clarithromycin [CLR], metronidazole [MTZ], and levofloxacin [LEV]) has exceeded the 15% threshold within all World Health Organization-monitored areas [9]. The same researchers also determined that the risk of therapeutic failures, compared with infections caused by antibiotic-sensitive *H. pylori* strains, increases 8-fold, 7-fold, and 2.5-fold when treating isolates resistant to LEV, CLR, and MTZ, respectively. Difficulties in achieving the therapeutic effect have a direct impact on the inclusion of *H. pylori* in the twelve most dangerous pathogens for which searching for new eradication methods is highly needed [10]. It is important to note that only CLR-resistant *H. pylori* strains are included on this list [10]. The degree of MTZ resistance determined in vitro, although very high in many countries around the world, does not correlate linearly with treatment efficacy [11]. This is most likely associated with the lack of gradient of oxido-reduction potential under laboratory conditions, which is a key factor responsible for the transformation of a prodrug into an effective antibiotic in *H. pylori* cells. Currently, bismuth salts therapy (bismuth subsalicylate, MTZ, tetracycline [TET], and proton pump inhibitors [PPIs]), with a degree of >80% eradication, enjoys great interest in areas with a high prevalence of antibiotic-resistant *H. pylori* strains [12]. Therefore, it is currently recommended as the first line of therapy [8]. Regardless of the therapeutic effectiveness of this formulation, there is still a need to search for alternative compounds active against *H. pylori*.

*H. pylori* is classically present as spirally-twisted rods, whereas its high heterogeneity contributes to the presence of various cell shapes, including straight or curved rods, elongated (filamentous) forms, or coccoid forms [13]. The occurrence of coccoid *H. pylori* forms was first described in 1991 [14]. In later years, the presence of this morphology was repeatedly confirmed, while its function was not established [15,16,17,18,19,20,21]. Initially, the production of this morphotype was thought to be an expression of cell death. This conclusion was drawn based on a loss of bacterial culturability during the morphological transition to coccoid forms. With the development of more sophisticated microbiological and genetic techniques, however, it began to be suggested that these cells are alive, although they have changed physiology. Morphological transformation into spherical forms by *H. pylori* is accompanied by a decrease in cell size and a drastic decrease in metabolic activity, which translates into a transition to a viable but non-culturable (VBNC) phenotype [20,22,23,24,25,26]. Despite this, there are reports indicating the possibility of producing diseases by spherical *H. pylori* forms [27,28,29,30,31,32]. Moreover, these forms have been shown to be able to avoid immune responses [33,34], promote carcinogenesis [31,35], and take part in therapeutic failures [30,32]. Additionally, Kadkhodaei et al. were able to obtain a culturable *H. pylori* strain occurring only as coccoids and, unlike the spiral-shaped parental strain, the former was characterized by mucus overproduction and resistance to all tested antibiotics [36]. These results suggest the importance of expanding awareness about the presence of spherical *H. pylori* forms and their impact on the activity of antimicrobial substances.

The current state of knowledge about the role of coccoid *H. pylori* forms is insufficient. Studies determining an activity of antimicrobial substances against *H. pylori* very often overlook the capacity of these bacteria to produce spherical forms. This mechanism, however, may have a vital function in reducing the effectiveness of antimicrobial therapies. Therefore, the purpose of this review was to gather information on the morphological transformation of *H. pylori* in the context of in vitro testing of antimicrobial compounds.

## 2. Review Strategy and Literature Included

The search for articles was performed using the keywords “*Helicobacter pylori*” OR “*H. pylori*” AND “antibacterial” OR “antimicrobial” in the Scopus database. The keywords did not include “coccoid forms” because it was noticed that this expression often did not appear in titles or keywords of articles. Only English-language papers published or accepted for publication between 2000 and 2019 were taken into account. In each publication, all literature references were reviewed, thus obtaining additional articles that did not appear during the primary screening. The initial search indicated the presence of 3139 articles. The first and most important step was to select only those publications in which both conditions were met: antimicrobial substances were tested against *H. pylori* and transformation to coccoid forms was noticed. In this way, 51 articles were obtained. The second step in the selection was to exclude articles in which substances were tested without determining a minimal inhibitory concentration (MIC) and/or minimal bactericidal concentration (MBC), one substance concentration and one time point were used, the effect of bacterial post-culture extracts against *H. pylori* was determined, and the activity of compounds was determined only against biofilm *H. pylori* forms. After applying the above-mentioned criteria, 32 articles being the core of the current review were obtained.

## 3. Results

### 3.1. Antibiotics and Proton Pump Inhibitors

**Figure 1:**
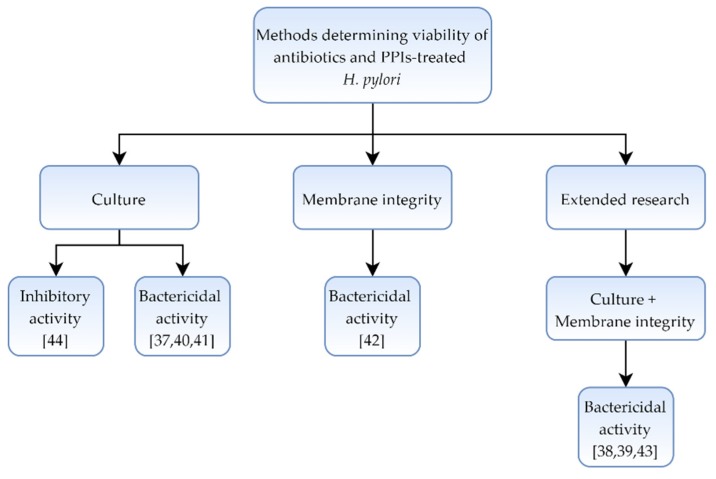
Diagram presenting the activity of classically used antibiotics and proton pump inhibitors (PPIs) against *Helicobacter pylori*.

In a collection of eight articles [37,38,39,40,41,42,43,44] showing a morphological effect of antibiotics and other substances classically used in *H. pylori* therapies, microscopic and culture methods were used. In four of them [38,39,42,43], different staining techniques and fluorescence analysis were additionally included (Table 1 and Figure 1). 

In the first article [37], dedicated to an activity of amoxicillin (AMX) and β-lactamase inhibitors, the ability of these substances to stimulate production of coccoid forms and separation of outer and inner membranes during 3–6 h of exposure was noticed.

In the next five analyzed articles [38,39,40,41,43], observations regarding a morphostructural effect of antibiotics come from publications primarily focusing on new compounds with a potential anti-*H. pylori* activity. Narayana et al. [38,39] showed that AMX within 24 h reduced the viability of *H. pylori* by 3 log when treated with MIC. This phenomenon was accompanied by an appearance of spherical forms and an increased permeability of cell membranes. In subsequent articles [40,41], it was observed that 72-h exposure of *H. pylori* to MICs of tested antibiotics (AMX, CLR, MTZ, and TET) resulted in a presence of numerous coccoid forms (>85%). The morphological effect of these antibiotics was significantly lower, although still present, when sub-MICs of CLR, TET, and MTZ were used. An amount of spherical forms after 72 h counted for <15% and 25–30% for ¼× MIC and ½× MIC, respectively. Formation of coccoid forms was the most intense for AMX and after 72 h was equal to 30% and 70% for ¼× MIC and ½× MIC, respectively. These observations are in line with the reports of Faghri et al. [42], who noticed that sublethal CLR and MTZ concentrations after 3 days of exposure have a relatively low potential to stimulate transformation of *H. pylori* into spherical forms (30–40%). This was in opposition to the very high pro-transformative potential of AMX (>95%). Furthermore, a one-day incubation of spherical *H. pylori* forms with 2× MIC of these antibiotics reduced the viability by 99.8% (CLR) and 62.6% (MTZ), while only 0.03% when exposed to AMX. The decreased sensitivity of coccoid forms to AMX was also recognized by Obonyo et al. [43]. Here, a 24-h treatment with MIC of this antibiotic completely lowered the viability of spiral forms, while the use of very high concentrations of AMX (20× MIC) reduced an amount of viable spherical forms only by 20%.

The last included article [44] determined an activity of omeprazole (a proton pump inhibitor) and found a direct relationship between the viability and number of coccoid forms. Exposure to ½× MIC for 3, 6, and 9 days contributed to the formation of 10%, 40%, and 90% of these morphological forms, respectively. In addition, an increase in the viability and transformation into spiral forms was obtained by removing omeprazole from the culture medium.

### 3.2. Plant Extracts and Plant-derived Compounds

**Figure 2:**
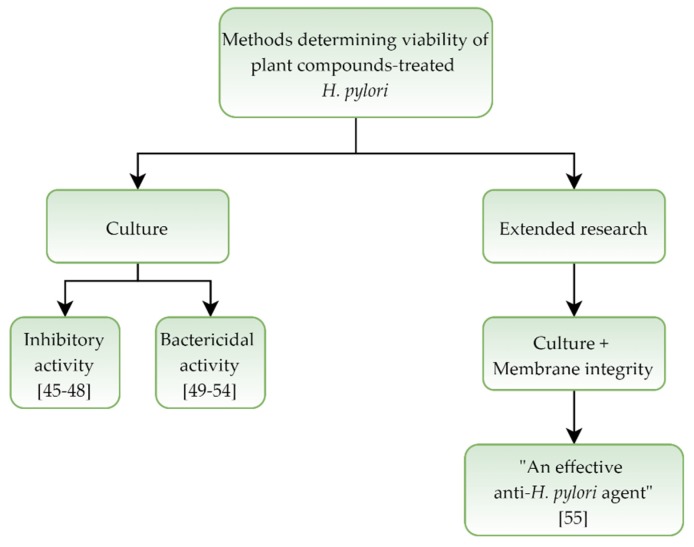
Diagram presenting activity of plant extracts and plant-derived compounds against *Helicobacter pylori.*

In a set of eleven studies [45,46,47,48,49,50,51,52,53,54,55] determining an activity and morphological effect of plant extracts and plant-derived compounds on *H. pylori*, four of them reported a bacteriostatic activity [45,46,47,48] and six indicated a bactericidal effect [49,50,51,52,53,54] of the tested compounds. In one case, the use of this classification was avoided [55]. In almost all (10 out of 11) basic techniques, microscopy and culture methods, were used. Only the one article has been expanded to include a number of fluorescence measurement techniques [55] (Table 2 and Figure 2).

Among the four studies [45,46,47,48] suggesting the bacteriostatic effect on *H. pylori*, in two a dominance of spherical forms was noted [46,48], in one the stimulation was low (33% after 48 h) [45], while in the last the production of spherical forms was not observed [47]. From these, an ability of the substance to completely reduce the viability was only demonstrated in the Ali et al. study [46], which took place after 6 h and 9 h for 2× MBC and MBC, respectively. 

The six studies indicated the bactericidal effect of the tested compounds on *H. pylori* [49,50,51,52,53,54]. A capacity to completely inhibit growth was only proven in half of them [49,51,52], which was achieved in a range of 18 h [49] to 72 h [51] with MICs. A relationship between the decrease in viability and number of spherical forms was particularly well illustrated in the Lee et al. article [51]. Here, a 48-h incubation with MIC (a 2 log viability decrease) and 2× MIC (a total viability reduction) of dehydrocostus lactone from *Magnolia sieboldii* leaves induced the formation of 49% and 94% of these morphological forms, respectively. In the remaining three articles [50,53,54], the decrease in viability of treated cells was different and ranged from 1 log after 6-h exposure to 2× MIC [53] to 3–4 logs after 12 h with a fractional inhibitory concentration (FIC) of the tested compounds [54]. In the case of two articles [53,54], an ability of the tested substances to induce cell destruction and create forms with degradative properties was indicated. 

Authors of the last analyzed article [55] interpreted the obtained results in the most careful manner, indicating that “daphnetin has a potential to be an effective anti-*H. pylori* agent”, despite the use of several techniques to determine the bacterial viability. Microorganisms treated with ½× MIC of daphnetin after 72 h had many morphostructural changes when observed by a scanning electron microscopy (SEM), but these observations were not confirmed using fluorescent techniques. However, it was recognized that DNA damages were induced.

### 3.3. Synthetic Compounds

**Figure 3:**
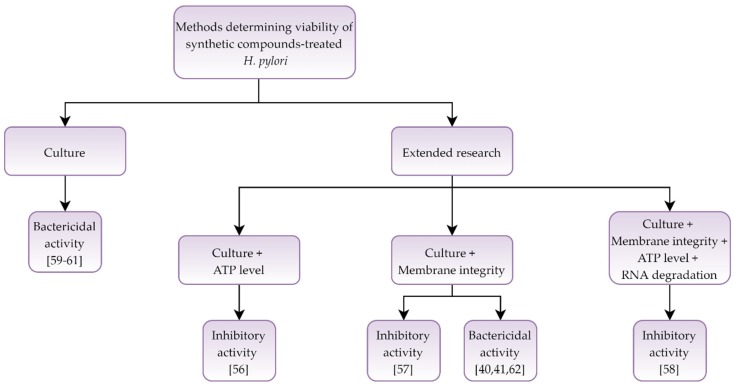
Diagram presenting activity of synthetic compounds against *Helicobacter pylori.*

In a collection of nine articles [40,41,56,57,58,59,60,61,62] devoted to the activity and morphological effect of synthetic compounds against *H. pylori*, three of the tested compounds were reported as bacteriostatic [56,57,58] and five marked as bactericidal [40,41,60,61,62]. In one article, the effect on bacterial cells was dependent on the agent used [59]. Most articles (5 out of 9) used microscopy, culture, and fluorescence methods to determine the morphology and viability [40,41,56,57,62], while one study was expanded to include molecular methods [58] (Table 3 and Figure 3).

The studies indicating the bacteriostatic effect of the tested substances [56,57,58] showed that a one-day incubation of bacteria with selected concentrations of each agent reduced the viability by only 1 log. Despite the low bactericidal potential, a stimulation of coccoid forms in all of them was noticed. Barry et al. [56] also proved that the effect of morphological variability was reversible. In the Chakraborti et al. study [58], despite many observations indicating physio-structural changes of bacterial cells (membrane damage, rRNA degradation, and ATP reduction), authors chose a high number of grown bacteria (the culturability) as the main parameter and interpreted polyethyleneimine functionalized zinc oxide particles as bacteriostatic against *H. pylori*. 

Another article [59] noted that polyoxometalates have a different activity against *H. pylori*, i.e., bactericidal for As_4_W_40_ and Sb_9_W_21_ or bacteriostatic for SiVW_11_. The effect of growth inhibition/reduction was directly correlated with an amount of coccoid forms. 

In the remaining five analyzed articles [40,41,60,61,62], the bactericidal activity of synthetic compounds was suggested. Four out of five studies showed a total reduction in the viability, while in one research a decrease only by 1 log after 24 h was noticed. In this case, the bactericidal effect was interpreted based on transmission electron microscopy (TEM) observations showing coccoid forms with broken cell membranes. The results of Dai et al. [60] and Kamoda et al. [61] seem to be problematic in the assessment. In the first case, high concentrations were used to achieve a complete bactericidal activity (8× MIC, 24 h) or morphostructural changes (64× MIC, 3 h) [60]. In the second, a discrepancy in results was observed, i.e., no change in membrane permeability using a fluorescence analysis (2× MBC, 10 h) and a suggestion of disintegration of cellular membranes during TEM observations (2× MBC, 8 h) [61]. In the last two included studies [40,41], a stimulating effect of the substances on the transformation of cells into spherical forms was noticed. The presence of coccoid morphotype, however, was interpreted as non-protective as a significant, concentration-dependent reduction in green/red fluorescence was observed.

### 3.4. Fatty Acids and Fatty Acid Derivatives

**Figure 4:**
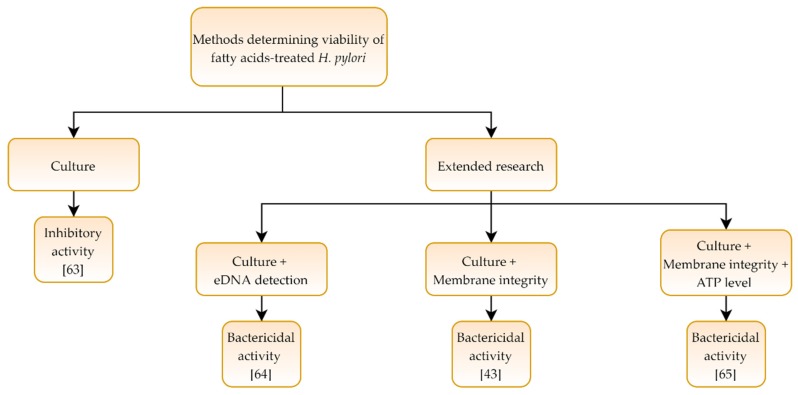
Diagram presenting activity of fatty acids and their derivatives against *Helicobacter pylori.*

In four articles [43,63,64,65] determining the effect of fatty acids and fatty acid derivatives on *H. pylori*, one research used microscopy and culture methods demonstrating a bacteriostatic nature of the tested compound [63], while other three studies were expanded to include fluorescent or molecular methods, and here the activity of the compounds was interpreted as bactericidal [43,64,65] (Table 4 and Figure 4). 

In the article suggesting the bacteriostatic activity of the tested fatty acid, a decrease in the viability by <2 log after 48-h exposure to MIC was observed [63]. Evaluation of morphology showed an existence of mixed populations composed of both coccoid and spiral forms. 

The remaining three included articles [43,64,65] suggested an existence of bactericidal properties of the compounds. Yonezawa et al. [64] noted that a 24-h incubation with MBC of sodium butyrate stimulated an appearance of spherical forms and accompanied release of membrane vesicles and eDNA. These mechanisms were, however, not reflected in the bacterial viability (<0.5 log reduction). The decrease in viability was seen after 48-h exposure to MBC (a 4-log reduction). The next article [65] noted a rapid decrease in the number of viable cells (a total bacterial destruction after 70 min with MIC of zinc linolenate), which was associated with a reduction in intracellular ATP, increase in the permeability of cellular membranes, vesiculation, and spherical forms appearance. In the last analyzed study [43], it was observed that the morphology of *H. pylori* affected the parameters required to kill the cells of this bacterium. Coccoid forms have been shown to require longer exposure and higher doses of liposomal linolenic acid (400 µg/mL, 24 h) than spiral forms (67 µg/mL, 0.5 h).

### 3.5. Peptides

**Figure 5:**
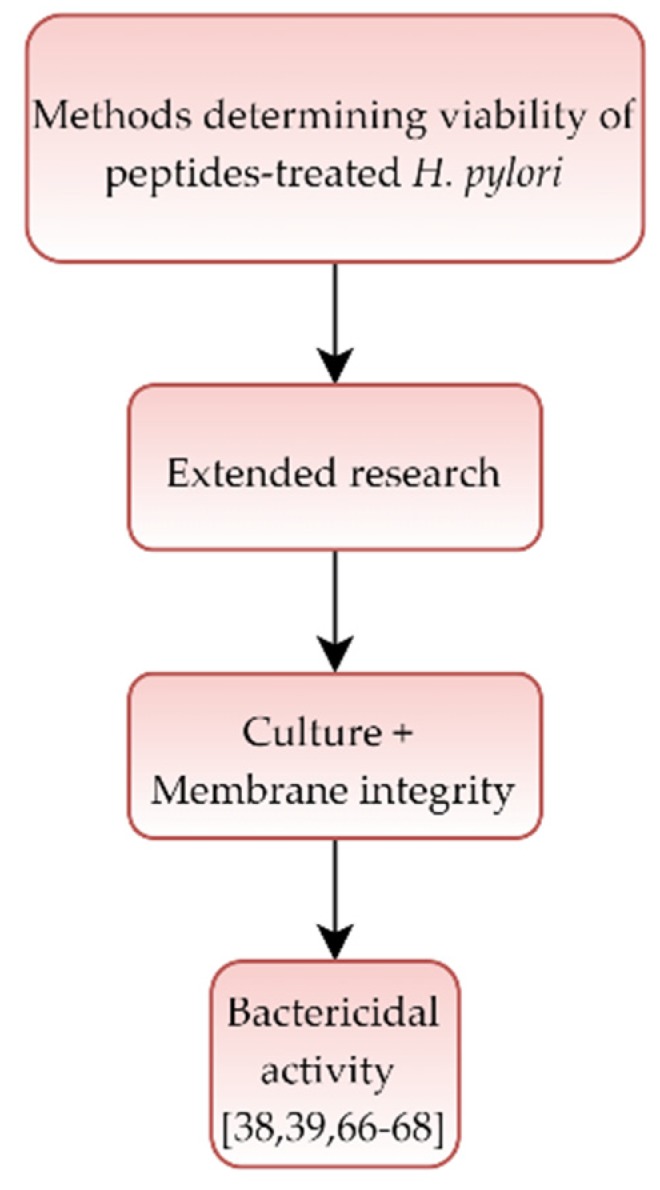
Diagram presenting activity of peptides against *Helicobacter pylori.*

The last five articles [38,39,66,67,68] concerned an activity and morphological effect of peptides against *H. pylori*. In all, a bactericidal effect was suggested, while the action of peptides on bacteria was demonstrated using microscopy, culture, and fluorescent methods (Table 5 and Figure 5). 

The three articles [38,39,66] noted that a use of peptides reduced the viability by 2 log with a time ranging from 3 h [39] to 24 h [38] with MICs. In all these articles, a TEM imaging and 1-N-phenylnaphthylamine (NPN) assays were used to observe an existence of coccoid forms with changed morphostructural properties and increased cell membrane permeability [38,39,66]. In the next research [67], it was noted that a 12-h incubation with 4× MIC of Cbf-K_16_ peptide reduced the viability only by 1 log. In this case, a reference was made to the destruction of cell structure, as demonstrated by TEM and various fluorescent methods. The last study [68] figured out a dynamic reduction in the bacterial viability with a total decrease observed after a 1-h incubation with MIC of the tested peptide. This phenomenon was accompanied by the appearance of spherical forms and a decrease in green fluorescence (SYTO9), characterizing cells with an intact cellular structure.

## 4. Discussion

The colonization of over 60% of people in the world by *H. pylori* and a direct involvement of this bacterium in the development of many gastrointestinal diseases, including cancers, have contributed to the high intensity of research conducted on epidemiology, diagnostics, and treatment of this microorganism [4,5,6,7]. Since 2000, more than 35,000 scientific reports on *H. pylori* have been published in PubMed, while only a fraction of them tried to find a link between the exposure to antimicrobial substances and *H. pylori* morphological changes.

In 20 out of 29 analyzed publications focusing on substances other than antibiotics, a bactericidal activity of the tested compounds was suggested (Table 2, Table 3, Table 4 and Table 5). Unfortunately, in most of these articles the morphological transformation of *H. pylori* was not included to show a potential risk of the spherical *H. pylori* forms development, but rather to demonstrate a morphostructural effect of the tested compounds. Only in two publications investigating the potential anti-*H. pylori* activity of new substances, the possibility of morphological reversion of this bacterium from spherical to spiral forms or higher tolerance of coccoid forms to the substances used was found (Table 3 and Table 4) [43,56]. Interestingly, among scientific reports raising the subject of an activity of antibiotics against *H. pylori* in the context of morphological changes, five out of eight showed the presence of such phenomena (Table 1) [40,41,42,43,44]. The reason for such a large difference may be caused by the fact that some of these results came from studies of newly tested substances, for which a desire to demonstrate the superiority over classically used antibiotics was noticed. Therefore, it seems that the obtained observations may not fully present the function of *H. pylori* morphological conversion as a protective mechanism against the action of antimicrobial substances.

### 4.1. Culture Methods in the Viability Assessing

**Figure 6:**
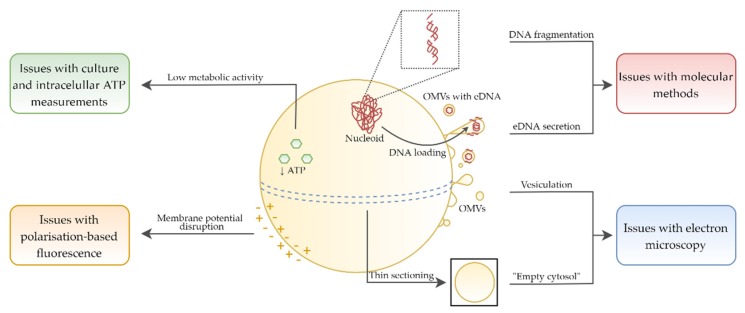
Physiological and morphological changes of coccoid *H. pylori* forms contributing to difficulties in the proper interpretation of viability during the antibacterial activity determination. Symbols: extracellular DNA (eDNA), outer membrane vesicles (OMVs).

In almost half of the publications not focusing on the antibiotics activity (14/29), the only research technique used in determining the viability of *H. pylori* was culture (Table 2, Table 3 and Table 4). Practically all of them concerned plant compounds (Table 2). The use of culture methods in assessing the viability of this bacterium is associated with a high risk of false-negative results [69]. When exposed to antimicrobial substances, this bacterium produces coccoid forms, which are most often (but not always) in the VBNC phenotype [26]. Thus, observations of a decline in the culturability may not be synonymous with a decrease in the viability. Lack of growth on culture media could be related to, e.g., induction of a state with a very low metabolic level, low cell density which cannot be detected by standard culture methods, or difficulties to resuscitate coccoid *H. pylori* forms in vitro (Figure 6) [70]. Hence, if other research techniques are not taken into account in assessing the viability of *H. pylori*, the possibility of omitting processes associated with the culturability loss and drawing erroneous conclusions regarding the viability of this bacterium is high.

### 4.2. Extending Techniques to Determine the Viability

In the remaining publications (15/29), research was expanded by molecular methods, fluorescence measurements, or bioluminescence analysis. The purpose of their inclusion was to determine an amount of eDNA or stability of bacterial genetic material, assess cell membrane permeability, and estimate ATP level, respectively. In addition, in some articles, observations using electron microscopy were used to determine the cell structure integrity. Consideration of the above-mentioned methods is very valuable for the reliability of obtained results, while each of them seems to have some limitations in the analysis of coccoid *H. pylori* forms (Figure 6).

#### 4.2.1. Amount of ATP in the Viability Assessment 

Estimating an amount of intracellular ATP (iATP) in *H. pylori* cells as an indicator of the viability of these bacteria appears to be problematic. It has been observed that coccoid *H. pylori* forms have changed physiology and the amount of iATP is over 100-fold to 2000-fold lower than in spiral forms (Figure 6) [20,71,72]. Moreover, the same studies excluded the presence of extracellular ATP (eATP) in culture media, indicating the compactness of cellular structures and suggesting that measuring the amount of eATP may be a better indicator of the viability of coccoid forms treated with antimicrobial substances.

It should be kept in mind that bacteria have an ability to convert ATP to polyphosphate, which constitute an alternative source of energy [73]. Although guanosine tetraphosphate (ppGpp), an alarmone, is responsible for the production of polyphosphates and transition of bacteria to persistent forms, these processes can also occur in the absence of this signal compound [74]. The first report indicating the ability of *H. pylori* to synthesize polyphosphate granules has been shown in 1993 [75], and these observations have then been confirmed later in many independent studies [19,23,24,71,76]. The presence of numerous polyphosphate granules was noticed in long-term incubated coccoid *H. pylori* forms (27% and 11% of spherical forms with polyphosphate granules after 45 days and 28 months, respectively) [71]. Based on these observations, it seems that counting the amount of polyphosphate granules of spherical *H. pylori* forms may be as important for determining a bioenergetic level as assessing the amount of iATP and eATP.

#### 4.2.2. Morphostructure Determined by TEM in the Viability Assessment

**Figure 7:**
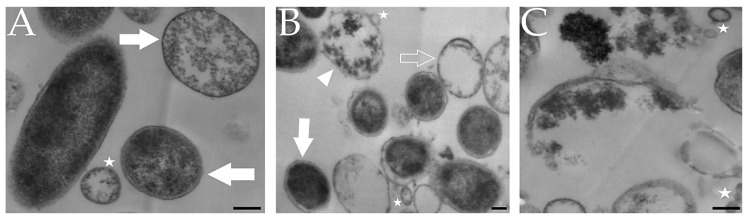
Representative photos of *H. pylori* cells taken with transmission electron microscopy (TEM). (**A**) Mixed population of spiral/rod-shaped and coccoid cells. (**B**) Population of coccoid cells in different physiological states. (**C**) Coccoid cell after degradation (cell lysis). Symbols: coccoid form with dense cytosol (white arrow), coccoid form with “empty cytosol” (empty arrow), irregularly- shaped coccoid form (white arrowhead), outer membrane vesicles (white stars). Scale bar is equal to 0.2 µm.

In some publications, electron microscopy was used to assess both the cell shape and morphostructural changes of *H. pylori*. The suggestion of the reduced integrity of the bacterial cell structure based on observations using transmission electron microscopy (TEM) is, however, at risk of over-interpretation (Figure 6 and Figure 7).

Research using *Escherichia coli* showed that a cell population consisting mainly of bacteria in the VBNC state had a lower number of ribosomes and a higher degree of protein oxidation than a population capable of a rapid resuscitation [77]. It was noticed that the reduction in ribosomes and DNA amount in VBNC *E. coli* cells gave the impression of an empty cytoplasm, while in both studies where this process was observed cell death was excluded [78,79]. In the first case by the lack of staining of such cells with propidium iodide [78], in the second case by the possibility to resuscitate them in culture media [79]. It is suggested that a very thin sectioning during TEM preparation may contribute to the apparent lack of small, highly condensed nucleoid and protein aggregates, giving the effect of empty cytosol (ghost cells) (Figure 6) [80]. Formation of these structures is promoted by the reduction in iATP and is crucial for the survival of microorganisms under stressogenic conditions [81].

Although these reports concerned microorganisms other than *H. pylori*, it cannot be ruled out that a very similar process may also take place in this bacterium. This hypothesis may be partly confirmed by observations indicating an ability to generate free hydroxyl radicals and the endogenous oxidative stress-dependent aggregation of proteins and DNA by coccoid *H. pylori* forms [82,83].

#### 4.2.3. Degradation of Genetic Material and eDNA Amount in the Viability Assessment 

Assessment of the stability of genetic material is another method determining the viability of spherical *H. pylori* forms being at risk of misinterpretation (Figure 6). In many research articles it has been noticed that the stability of their DNA and RNA is lower than in spiral forms [15,21,82,84,85]. In coccoid forms, a non-random fragmentation of genetic material occurs, while the amount of DNA and RNA is several times lower compared to spiral forms (Figure 6). Therefore, an observation of the genetic material disintegration of *H. pylori* may be the result of morphological transformation into spherical forms and not necessarily a genotoxic effect of tested substances.

An additional aspect worth mentioning is the assessment of eDNA amount as an indicator of the cellular membrane permeability. Rossella et al. have shown that in a process of outer membrane vesicles (OMVs) secretion by *H. pylori*, eDNA is released (Figure 6) [86,87]. This, however, is a physiological process that can play an important role in the *H. pylori* survival. Protective function of eDNA is associated with a participation in a horizontal gene transfer [88,89] and the maintenance of biofilm stability [86]. In addition, it has been suggested that the release of OMVs during transformation of *H. pylori* from a spiral to a coccoid form is associated with a reduction in cell size [24]. The similar process was also detected in VBNC *Vibrio parahaemolyticus* cells and was related to a starvation-dependent reduction in metabolic activity [90].

#### 4.2.4. Fluorescence Determination in the Viability Assessment 

Nowadays, the least information regarding a possibility of misinterpretations of the *H. pylori* viability concerns fluorescence methods determining cell membrane permeability. For this purpose, SYTO9/propidium iodide (so called Live/Dead) and NPN staining are most commonly used in *H. pylori* studies [69]. It seems that a basic criterion for cells with the VBNC phenotype, an integrity of cellular structures, contributes to an effective determination of the viability of coccoid *H. pylori* forms using the aforementioned methods [80]. It should be borne in mind, however, that morphological transition may somehow affect the adsorption or retention capacity of some fluorescent dyes. Since the loss of membrane potential has been seen in coccoid *H. pylori* forms, fluorescent dyes determining this parameter may have limited use in assessing their viability (Figure 6) [15]. Research in this area is undoubtedly needed.

### 4.3. Difficulties in Determining the Viability of H. pylori in the Context of Applied Research Techniques

The reservations presented above are reflected in the heterogeneity of the results obtained in some of the analyzed articles, in which several research methods determining the viability of *H. pylori* were used. 

In the study by Wang et al. [55], using microscopic observations and PCR, the existence of morphostructural changes and DNA degradation were noticed. On the other hand, bacterial cells maintained a high level of culturability and a high ratio of green/red fluorescence, indicating their high viability. In the article by Chakraborti et al. [58], a high number of culturable cells was also observed, while other techniques indicated a different situation, i.e., rRNA degradation and reduced level of iATP. In the study by Kamoda et al. [61], a total decrease in the culturability and morphostructural changes using TEM were noticed, however, a high green/red fluorescence ratio was still present.

The indication of the above publications in no way detracts from the quality of these results. On the contrary, it illustrates well the difficulties faced by scientists involved in analyzing the viability of antimicrobials-treated *H. pylori*. These seem to be, at least in part, associated with the presence of coccoid *H. pylori* forms, which are characterized by changed physiology and morphostructure in relation to spiral forms (Figure 6).

### 4.4. Future Research Determining the Viability of H. pylori

Modern research techniques assessing the viability of microorganisms, which are currently at the stage of validation, may in the future prove to be very helpful in determining the viability of coccoid *H. pylori* forms [70]. Promising techniques include a measurement of microbial heat flow, molecular viability testing (MVT) using an assessment of rRNA precursors production, a measurement of de novo synthesized ATP, or viability PCR (vPCR) using modified quantitative PCR. 

Until now, none of these techniques have been used to determine the viability of *H. pylori*, while the results of research on other microorganisms are optimistic. For example, the use of vPCR and MVT has been very helpful in distinguishing VBNC from dead cells of *Pseudomonas aeruginosa* treated with antibiotics [91]. The use of vPCR has also been successfully used in the detection of VBNC *Campylobacter* cells, bacteria closely related to *Helicobacter* [92]. Some hope in assessing the viability of coccoid *H. pylori* also gives the measurement of de novo synthesized ATP. It has been observed that exposure of these morphological forms to human erythrocyte lysate increased an ATP amount in spherical *H. pylori* forms by 12–150 times [71]. Hence, the de novo measurement of synthesized ATP could potentially distinguish between coccoid *H. pylori* forms being in the VBNC state from dead cells [93].

### 4.5. Implications for Scientists and Clinicians

From the scientific point of view, it seems important to answer the question: Which of these substances are the most effective against *H. pylori*? The heterogeneity of the results of the research presented in this review makes it difficult to clearly judge this issue. Based on a comparison of the level of bactericidal activity, speed of action, and an ability to act on both morphological forms of *H. pylori* (spiral and coccoid), one can be tempted to select representatives from each group of compounds: essential oils [46] and propolis components [54] (plant compounds); SQ109 [62], 3-bromopyruvate [40] and sertraline [41] (synthetic compounds); linolenic acid and its derivatives [43,65] (fatty acids), and (PGA)_m_-*r*-(PHLG-MHH)_n_ [68] (peptides). It is suggested that promising results obtained in vitro for planktonic *H. pylori* forms for these substances should be verified in vivo and for biofilm forms of these bacteria.

From the clinical point of view, it seems important to answer the following questions: How can the presence of coccoid *H. pylori* forms affect the therapeutic effect and choice of drugs? How can these morphological forms affect the diagnosis of the bacterium? Considering the first issue, based on a review of the literature on antibiotics, it appears that AMX is highly effective against spiral *H. pylori* forms and the morphological transformation of AMX-exposed bacteria has no protective effect [37,38,39,42]. A different case is observed when treating the pre-formed spherical forms of *H. pylori*, for which very low sensitivity to this antibiotic was demonstrated [42,43]. The pre-formed coccoid forms of *H. pylori* may be present in patients who have undergone multiple antibiotic therapies, hence the use of other antibiotics, e.g., CLR or MTZ, which have higher activity relative to coccoid *H. pylori* forms, seems reasonable [42]. On the other hand, the diagnostic aspect of spherical *H. pylori* forms should also be considered. Because these forms are nonculturable and have a low degree of metabolic activity, their presence may be associated with an increased risk of false-negative results [94,95,96]. This is especially important in patients after *H. pylori* eradication or those receiving PPIs for a long term. At this point, the importance of biopsy analysis for the coccoid *H. pylori* forms presence and modification of therapy based on patient’s treatment history are indicated.

## 5. Conclusions

Based on a review of the literature from the last 20 years regarding the morphological variability of *H. pylori*, in studies determining the activity of new antimicrobial substances it is recommended to:

- Apply several methods determining the viability of *H. pylori.*
- Search for new, more accurate techniques determining the viability of *H. pylori.*
- Test the activity of substances against preformed coccoid *H. pylori* forms (regardless of studies on spiral forms).
- Use coccoid *H. pylori* forms, not treated with antimicrobial substances, as a control of the activity of substances against these morphological forms (without reference to spiral forms characterized by different physiology).

## Figures and Tables

**Table 1:** Activity and morphological effect of classically used antibiotics and proton pump inhibitors on *H. pylori*.

Compound	*H. pylori* Strain(s)	Technic Determining Morphology	Technic Determining Viability	Main Results Covering the Morphological Transformation of *H. pylori*	Authors Interpretation	Bibliography
SUL TAZ CLV AMX AMX + CLV	ATCC 43504	Light microscopy, TEM	Culture	- Viability ↓ <1 log, 6 h, 10× MIC (CLV, SUL, and TAZ) - Coccoid forms with CLV, SUL, and TAZ (6 h, 10× MIC) - Viability ↓ 1 log, 3 h, MIC (AMX, AMX+CLV) - Coccoid forms with AMX and AMX+CLV (3 h, MIC) - Separation of membranes for all antimicrobials (3–6 h, MIC)	Bactericidal activity	[37]
AMX	ATCC 43504	TEM	Culture, Fluorescence (membrane integrity)	- Viability ↓ (3 log, 24 h, MIC) - Coccoid forms (2 h, MIC) - Membrane permeabilization (2 h, MIC)	Bactericidal activity	[38,39] *
CLR TET MTZ AMX	Tx30a	Light microscopy	Culture	- Total viability ↓ for all antibiotics (72 h, MIC) - ≥85% of coccoid forms for all antibiotics (72 h, MIC) - Approx. 30% (¼× MIC) and 70% (½× MIC) of coccoid forms with AMX (72 h) - <15% (¼× MIC) and 25–30% (½× MIC) of coccoid forms with CLR, TET, or MTZ (72 h)	Bactericidal activity, AMX is the strongest inducer of a spiral-to-coccoid form transformation	[40,41] *
AMX MTZ CLR	ATCC 700392	Light microscopy	Flow cytometry (membrane integrity)	- Approx. 30%, 40%, and 95% of coccoid forms with CLR, MTZ, and AMX, respectively (72 h, ½× MIC) - Viability ↓ of coccoid forms by 99.8%, 62.6%, and 0.03% with CLR, MTZ, and AMX, respectively (24 h, 2× MIC)	Bactericidal activity, lower susceptibility of coccoid forms to antibiotics (especially to AMX)	[42]
AMX	SS1	SEM	Culture, Fluorescence (membrane integrity)	- Total viability ↓ of spiral forms (24 h, MIC) - Viability ↓ of coccoid forms only by 20% (24 h, 20× MIC)	Bactericidal activity, coccoid forms are more tolerant to AMX than spiral forms	[43] *
Omeprazole	Clinical isolates	Light microscopy	Culture	- Viability ↓ was directly associated with an amount of coccoid forms - 10%, 40%, and 90% of coccoid forms after 3, 6, and 9 days, respectively (½× MIC) - Viability and spiral shape were recovered after 9–12 days of the omeprazole removal	Inhibitory activity with the reversibility of morphology	[44]

Symbols: decrease (↓), increase (↑). Abbreviations: clavulonate (CLV), sulbactam (SUL), tazobactam (TAZ), amoxicillin (AMX), clarithromycin (CLR), metronidazole (MTZ), tetracycline (TET), proton pump inhibitors (PPIs), scanning electron microscopy (SEM), transmission electron microscopy (TEM), minimal inhibitory concentration (MIC), minimal bactericidal concentration (MBC). * In studies marked with asterisks observations regarding antibiotics come from publications primarily focusing on new compounds with a potential anti-*H. pylori* activity.

**Table 2:** Activity and morphological effect of plant extracts and plant-derived compounds on *H. pylori*.

Compound	*H. pylori* Strain(s)	Technic Determining Morphology	Technic Determining Viability	Main Results Covering the Morphological Transformation of *H. pylori*	Authors Interpretation	Bibliography
Cranberry extract	NCTC 11637	SEM	Culture	- Viability ↓ (2 log, 48 h, MIC) - 33% of coccoid forms (48 h, MIC)	Inhibitory activity	[45]
Eugenol Cinnamaldehyde	ATCC 700392	Light microscopy	Culture	- Total viability ↓ (9 h, MBC or 6 h, 2× MBC) - Viability ↓ (>3 log, 4 h, 2× MBC) - 70% of coccoid forms (4 h, 2× MBC)	Inhibitory activity	[46]
Boropinol A	B128	SEM	Culture	- Viability ↓ (several times lower OD, 48 h, MIC) - Spiral forms with shorter length and blebbing (24 h, MIC)	Inhibitory activity	[47]
7-O-Butylnaringenin	ATCC 700392	SEM	Culture	- Viability ↓ (<3 log, 24 h, MIC) - Coccoid-like cells with irregular shapes (24 h, MIC)	Inhibitory activity	[48]
β-ACPM	ATCC 43504	TEM	Culture	- Total viability ↓ (18 h, MIC) - Coccoid forms with blebbing, detachment of the outer cell membrane, and membrane invaginations (24 h, MIC)	Bactericidal activity	[49]
Methyl gallate Benzoic acid PGG Paeonol	ATCC 43504	Light microscopy	Culture	- Viability ↓ (1–2 log, 48 h, MIC) - Viability ↓ (≥5 log, 48 h, 2× MIC) - 43–52% of coccoid forms (48 h, MIC) - 74–91% of coccoid forms (48 h, 2× MIC)	Bactericidal activity	[50]
DCL from *Magnolia sieboldii* leaves	ATCC 43504	SEM	Culture	- Total viability ↓ (36 h, 2× MIC) - Viability ↓ (2 log, 48 h, MIC) - 49% and 94% of coccoid forms (48 h, MIC and 2× MIC, respectively)	Bactericidal activity	[51]
Isocoumarin paepalantine	ATCC 43504	SEM	Culture	- Total viability ↓ (72 h, MIC) - Spiral forms (72 h, ½× MIC) - Coccoid forms (72 h, MIC)	Bactericidal activity	[52]
Patchouli alcohol	NCTC 11637	SEM, TEM	Culture	- Viability ↓ (1 log, 6 h, 2× MIC) - Mix of both spiral and coccoid forms (72 h, sub-MICs) - Coccoid forms with blebbing, cell wall damage, and lysis of the cytoplasmic membrane (1 h, MIC or 2 h, 2× MIC)	Bactericidal activity	[53]
Chrysin (CH) Pinocembrin (P) Galangin (G) Caffeic acid (CA)	ATCC 43504	TEM	Culture	- Viability ↓ (4 log, 12 h, FIC of CH+P) - Viability ↓ (3 log, 12 h, FIC of G+CA) - Ghost (degenerative) cells with coccoid-like shape, membrane damage, and membrane vesicles formation (12 h, FIC of CH+P or G+CA)	Bactericidal activity	[54]
Daphnetin	ATCC 43504	SEM, TEM	Culture, Fluorescence + CLSM + Flow cytometry (membrane integrity)	- Spiral forms (72 h, ¼× MIC) - Single coccoid forms (72 h, ½× MIC) - Budding, rough outer membrane, peculiar detachments between membrane and cytoplasm, and numerous membrane vesicles (72 h, ½× MIC) - No membrane permeability and depolarization (24 h, ½× MIC) - DNA damage (24 h, ½× MIC)	Daphnetin has a potential to be an effective anti-*H. pylori* agent	[55]

Symbols: decrease (↓), increase (↑). Abbreviations: Beta-artecyclopropylmether (β-ACPM), dehydrocostus lactone (DCL), 1,2,3,4,6-penta-O-galloyl-β-D-glucopyranose (PGG), confocal laser scanning microscopy (CLSM), scanning electron microscopy (SEM), transmission electron microscopy (TEM), minimal inhibitory concentration (MIC), minimal bactericidal concentration (MBC), fractional inhibitory concentration (FIC), optical density (OD).

**Table 3:** Activity and morphological effect of synthetic compounds on *H. pylori*.

Compound	*H. pylori* Strain(s)	Technic Determining Morphology	Technic Determining Viability	Main Results Covering the Morphological Transformation of *H. pylori*	Authors Interpretation	Bibliography
DFMO	SS1	Light microscopy, TEM	Culture, Luminescence (ATP level)	- Viability ↓ (<1 log, 24 h, MIC) - Coccoid forms (6 h, MIC) - Reversion from coccoid to spiral forms (6 h after the DFMO removal) - ↑ iATP level (0–12 h, MIC), no difference later (24 h, MIC)	Inhibitory activity with the reversibility of morphology	[56]
Niclosamide	ATCC 49503	SEM	Culture, Fluorescence (membrane integrity)	- Viability ↓ (approx. 1.5 log, 24 h, 4× MIC) - Coccoid forms produced in a dose-dependent manner (3 h, MIC to 8× MIC) - No membrane permeabilization (1 h, 64× MIC)	Inhibitory activity	[57]
Polyethyleneimine functionalized zinc oxide	ATCC 700392	SEM, TEM	Culture, Luminescence (ATP level), qRT-PCR (RNA degradation), Fluorescence (membrane integrity)	- Viability ↓ (1 log, 24 h, MIC) - 10% (0.5 h, MIC) and 33% (3 h, MIC) of coccoid forms - Membrane damage (3 h, MIC) - ↓ iATP level (3 h, MIC) - Degradation of rRNA (time-dependent, MIC)	Inhibitory activity	[58]
Polyoxometalates: As_4_W_40_ Sb_9_W_21_ SiVW_11_	ATCC 43504	SEM	Culture	- Total viability ↓ for As_4_W_40_ and Sb_9_W_21_ (10× MIC, 24 h and 12 h, respectively) - Stable viability for SiVW_11_ (10× MIC, 24 h) - Intensive formation of coccoids for As_4_W_40_ and Sb_9_W_21_ (36 h, > MICs) - Weak formation of coccoids for SiVW_11_ (36 h, > MICs)	Bactericidal (As_4_W_40_ and SB_9_W_21_) or bacteriostatic (SiVW_11_) activity	[59]
NE-2001	ATCC 43504	TEM	Culture	- Viability ↓ (1 log, 24 h, MIC) - Coccoid forms with an intact membrane (6 h, ½× MIC) - Ghost (degenerative) cells with coccoid-like shape with a loosening of the outer membrane (6 h, MIC)	Bactericidal activity	[60]
TG44	ATCC 43504	SEM, TEM	Culture	- Total viability ↓ (24 h, 8× MIC) - Viability ↓ (<1 log, 24 h, MIC to 4× MIC) - Spiral forms with membrane vesicles around (3 h, ½× MIC to 4× MIC) - Coccoid forms with blebbing and outer membrane detachment (3 h, 64× MIC)	Bactericidal activity	[61]
SQ109	G27	TEM	Culture, Fluorescence (membrane integrity)	- Total viability ↓ (8 h, 2× MBC) - Coccoid forms (98–99%) with disintegration of the inner membrane (8 h, 2× MBC) - Ghost (degenerative) cells (1–2%) (8 h, 2× MBC) - No membrane disruption using fluorescence analysis (10 h, 2× MBC)	Bactericidal activity	[62]
3-Bromopyruvate	Tx30a J99	Light microscopy, SEM	Culture, Fluorescence (membrane integrity)	- Total viability ↓ (6 h, 4 h, and 2 h for MIC, 2× MIC, and 4× MIC, respectively) - 33.5% (Tx30a) and 57.5% (J99) of coccoid forms (2 h, 4× MIC) - 97–98% of coccoid forms for both (24 h, 4× MIC) - ↓ green/red fluorescence (time-dependent, MIC to 4× MIC)	Bactericidal activity	[40]
Sertraline	Tx30a J99	Light microscopy, SEM	Culture, Fluorescence (membrane integrity)	- Total viability ↓ (8 h, 4× MIC for J99 or 24 h, 4× MIC for Tx30a) - Viability ↓ (2–4 log, 24 h, MIC) - > 85% of coccoid forms (8 h, 4× MIC for J99 or 24 h, 4× MIC for Tx30a) - ↓ green/red fluorescence (time-dependent, MIC to 4× MIC)	Bactericidal activity	[41]

Symbols: decrease (↓), increase (↑). Abbreviations: difluoromethylornithine (DFMO), intracellular ATP (iATP), ribosomal RNA (rRNA), scanning electron microscopy (SEM), transmission electron microscopy (TEM), quantitative reverse transcription polymerase chain reaction (qRT-PCR), minimal inhibitory concentration (MIC), minimal bactericidal concentration (MBC).

**Table 4:** Activity and morphological effect of fatty acids and their derivatives on *H. pylori*.

Compound	*H. pylori* Strain(s)	Technic Determining Morphology	Technic Determining Viability	Main Results Covering the Morphological Transformation of *H. pylori*	Authors Interpretation	Bibliography
Docosahexaenoic acid	ATCC 700392, SS1	SEM	Culture	- Viability ↓ (<2 log, 48 h, MIC) - Mixed population of spiral and coccoid forms (48 h, MIC)	Inhibitory activity	[63]
Sodium butyrate	TK1402	SEM	Culture, PCR (eDNA detection)	- Stable viability (24 h, MBC) - Viability ↓ (4 log, 48 h, MBC) - Coccoid forms with blebs and cell envelope alternations (24 h, MBC) - ↑ eDNA amount (24 h, MBC)	Bactericidal activity	[64]
Zinc linolenate	G27	TEM	Culture, Luminescence (ATP level), Fluorescence (membrane integrity)	- Total viability ↓ (70 min, MIC) - Coccoid forms with membrane detachments, cytoplasmic content leakage, and vesiculation (2 h, MIC) - Membrane permeabilization (2 h, MIC) - ↓ iATP (2 h, MIC) and ↑ eATP (2 h, MIC)	Bactericidal activity	[65]
Liposomal linolenic acid	SS1	SEM	Culture, Fluorescence (membrane integrity)	- Total viability ↓ influenced by the morphology - MBC was higher for coccoid forms than spiral forms and killed cells more slowly (24 h, 400 µg/mL vs. 0.5 h, 67 µg/mL) - Disruption of bacterial membranes and cell clusters formation (0.5 h, MBC)	Bactericidal activity with coccoid forms being more tolerant	[43]

Symbols: decrease (↓), increase (↑). Abbreviations: extracellular DNA (eDNA), extracellular ATP (eATP), intracellular ATP (iATP), scanning electron microscopy (SEM), transmission electron microscopy (TEM), polymerase chain reaction (PCR), minimal inhibitory concentration (MIC), minimal bactericidal concentration (MBC).

**Table 5:** Activity and morphological effect of peptides on *H. pylori*.

Compound	*H. pylori* Strain(s)	Technic Determining Morphology	Technic Determining Viability	Main Results Covering the Morphological Transformation of *H. pylori*	Authors Interpretation	Bibliography
C_12_K-2β_12_	G27	TEM	Culture, Fluorescence (membrane integrity)	- Viability ↓ (>2 log, 8 h, 3× MIC) - Membrane permeabilization (8 h, 3× MIC) - Ghost (degenerative) cells with coccoid-like shape, blebbing, vesicularization, and loosening of the outer membrane (16 h, 3× MIC)	Bactericidal activity	[66]
Epinecidin-1	ATCC 43504	TEM	Culture, Fluorescence (membrane integrity)	- Viability ↓ (2 log, 12 h, 2× MIC or 2 log, 24 h, MIC) - Membrane permeabilization (6 h, MIC) - Ghost (degenerative) cells with coccoid-like shape, pore formation, blebbing, vesicularization, and loosening of the outer membrane (8 h, MIC)	Bactericidal activity	[38]
Tilapia Piscidin 4	ATCC 43504	TEM	Culture, Fluorescence (membrane integrity)	- Viability ↓ (2 log, 3 h, MIC or 3 log, 3 h, 2× MIC) - Membrane permeabilization (6 h, MIC) - Ghost (degenerative) cells with coccoid-like shape, micellization, and loosening of the outer membrane (2 h, MIC)	Bactericidal activity	[39]
Cbf-K_16_	SS1	TEM	Culture, Fluorescence + CLSM + Flow cytometry (membrane integrity)	- Viability ↓ (0.5 log, 8 h, 4× MIC or 1 log, 12 h, 4× MIC) - Ghost (degenerative) cells with coccoid-like shape, separation of membranes, and a total loss of cytoplasmic content (8 h, 4× MIC) - Membrane permeabilization (time-dependent, 4× MIC)	Bactericidal activity	[67]
(PGA)_m_-*r*-(PHLG-MHH)_n_	SS1	SEM	Culture, Fluorescence (membrane integrity)	- Total viability ↓ in pH = 3 (1 h, MIC) - Coccoid forms with membrane damage in pH = 3 (0.5 h, MIC) - Membrane permeabilization in pH = 3 (1 h, MIC)	Bactericidal activity in acidic environment	[68]

Symbols: decrease (↓), increase (↑). Abbreviations: confocal laser scanning microscopy (CLSM), scanning electron microscopy (SEM), transmission electron microscopy (TEM), minimal inhibitory concentration (MIC), minimal bactericidal concentration (MBC).
